# Correlations between primary tumour location, biomarkers of inflammation and lung injury, and postoperative pulmonary complications in patients underwent laparoscopic colorectomy: a propensity score matched analysis of 300 patients

**DOI:** 10.3389/fimmu.2025.1546167

**Published:** 2025-01-30

**Authors:** Hui-xian Li, Li Che, Yuan Li, Tai-hang Wang, Fang-di Min, Lei Xu, Meng Wang, Zhao-xu Zheng, Shi-ning Qu, Fei Wang, Wei Tang, Shi-jing Wei, Yu-lin Sun, Hui Zheng, Tao Yan

**Affiliations:** ^1^ Department of Anesthesiology, National Cancer Center/National Clinical Research Center for Cancer/Cancer Hospital, Chinese Academy of Medical Sciences and Peking Union Medical College, Beijing, China; ^2^ Department of Cardiology, Central Hospital of Dalian University of Technology, Dalian, China; ^3^ Department of Colorectal Surgery, Sun Yat-Sen University Cancer Center, Guangzhou, China; ^4^ Department of Colorectal Surgery, National Cancer Center/National Clinical Research Center for Cancer/Cancer Hospital, Chinese Academy of Medical Sciences and Peking Union Medical College, Beijing, China; ^5^ Department of Intensive Care Unit, National Cancer Center/National Clinical Research Center for Cancer/Cancer Hospital, Chinese Academy of Medical Sciences and Peking Union Medical College, Beijing, China; ^6^ Office of Cancer Screening, National Cancer Center/National Clinical Research Center for Cancer/Cancer Hospital, Chinese Academy of Medical Sciences and Peking Union Medical College, Beijing, China; ^7^ Department of Diagnostic Radiology, National Cancer Center/National Clinical Research Center for Cancer/Cancer Hospital, Chinese Academy of Medical Sciences and Peking Union Medical College, Beijing, China; ^8^ State Key Laboratory of Molecular Oncology, National Cancer Center/National Clinical Research Center for Cancer/Cancer Hospital, Chinese Academy of Medical Sciences and Peking Union Medical College, Beijing, China

**Keywords:** colorectal cancer, Trendelenburg position, inflammatory biomarker, lung injury, postoperative pulmonary complications

## Abstract

**Introduction:**

The impact of distinct primary colorectal cancer (CRC) sites on lung injury and complications remains largely unexplored, despite the palpable differences in surgical positions, procedures, and the resulting mechanically induced respiratory pressures at each site.

**Materials and methods:**

This study employed a forwards-looking approach utilising the propensity score matching (PSM) method; 300 patients with pathological CRC after laparoscopic surgery from April 2019 to May 2023 were enrolled. Two categories were bifurcated based on their surgical locations: the rectosigmoid colon (RSC) group and the descending/ascending colon (DAC) group, with a 2:1 ratio. The occurrence of postoperative pulmonary complications (PPCs) within a 30-day postoperative period was meticulously evaluated. Additionally, assessments have been performed for plasma biomarkers of immune response dynamics and lung injury (plasma soluble advanced glycation end-product receptor [sRAGE], angiopoietin-2 [ANG-2], interleukin-1β/6 [IL-1β/IL-6]) and other parameters.

**Results:**

Although the increase in postoperative lung epithelial damage, as indicated by the plasma sRAGE levels, was significant in the RSC group (DAC vs. RSC; 1029.6 [576.8–1365.2] vs. 1271.6 [896.3–1587.6]; odds ratio=0.999; 95% CI: 0.998 to 1.000; P=0.007), a significantly increased percentage of PPCs was observed in the DAC group (DAC vs. RSC; hazard ratio=1.669; 95% CI, 1.141 to 2.439; P=0.008). A univariate Cox proportional hazards model revealed that sRAGE, ANG-2, IL-1β, and IL-6 levels were not correlated with the incidence of time-to-PPCs across the two cohorts (P>0.05). Propensity score-weighted Cox regression and causal mediation analysis further demonstrated that the DAC site directly affected the incidence of PPCs, regardless of the other baseline confounders and clinical covariates related to the tumour site and PPCs.

**Conclusion:**

The primary site of CRC is an independent predictor of the development of PPCs. Despite the steep Trendelenburg position of the RSC group inciting more pulmonary stress, inflammation and lung epithelial injury, as indicated by higher sRAGE, it demonstrated a lower PPCs occurrence relative to its DAC counterpart, with a slightly inclined or reversed Trendelenburg position. None of the plasma biomarkers of inflammation or lung injury indicated sufficient prognostic value for PPCs.

## 1 Introduction

Postoperative pulmonary complications (PPCs), the second most recurrent complication in general surgical operations (1, 2), are intrinsically associated with perioperative morbidity and mortality (3–5). These complications are particularly predisposed to manifest during major abdominal surgeries (both laparotomy and laparoscopy) (6), with incidence rates ranging from 3–30% (3, 7, 8). This variability in incidence primarily arises from the heterogeneity in the definitions of PPCs (9), surgical/anaesthetic methodologies, and patient populations enrolled. A comprehensive review of the American College of Physicians demonstrated that a composite of pneumonia and respiratory failure defined PPCs in nearly 60% of the 16 preceding publications spanning over a quarter of a century (10). However, the European Perioperative Clinical Outcome (EPCO) consortium proposed that PPCs encompass respiratory failure, acute respiratory distress syndrome (ARDS), aspiration pneumonitis, pneumonia, respiratory infection, atelectasis, pleural effusion, pneumothorax, bronchospasm, and pulmonary embolus (11). Several studies have also incorporated lung injury and the subclinical stage of microatelectasis as PPCs (12–14).

Colorectal cancer (CRC), the most frequently observed type of abdominal tumour, represents the second highest incidence rate of 1,926,118 new cases (9.6% morbidity) and 903,859 deaths (9.3% mortality) globally in 2022 (15). CRC has a diverse global geographical distribution, with the majority of CRCs in Asia manifesting as rectal cancers, constituting more than 50% of the total cases. In contrast, in Europe and North America, rectal cancers represent less than 40% of all (16, 17). Given that the overwhelming majority of CRC patients are treated with laparoscopic procedures, the pneumoperitoneum pressure may amplify the skew in the ventilation−perfusion ratio and the generation of pulmonary atelectasis via enhanced transpulmonary pressure and worsened lung compliance, which can promote lung injury and PPCs even in patients with otherwise healthy lungs (18–20).

Notably, CRC can occur in disparate anatomical locations, including the ascending, transverse, descending and sigmoid colon, and rectum (inclusive of the anus). Thus, the surgical positions utilised in treating CRC are contingent upon the specific site of the disease. Rectosigmoid colectomy (RSC) necessitates a steep Trendelenburg position, whereas ascending and descending colectomy (DAC) merely requires a slightly inclined or reverse Trendelenburg position. Notably, the steep Trendelenburg position may induce substantial diaphragm displacement, haemodynamic compromise, reduced functional residual capacity, augmented pulmonary stress facilitating atelectasis, and compromised oxygenation (21–23). The integration of a large-angle Trendelenburg position along with pneumoperitoneum has also been verified to exacerbate more deterioration of the respiratory system, which can facilitate atelectasis and obstruct oxygenation and related inflammation and lung injury (24). In light of these factors, the primary CRC site, which involves different surgical positions, appears to be a crucial determinant of lung injury and subsequent PPCs incidence.

Despite extensive studies on predictive risk factors for preventing PPCs, including age, surgery type, preoperative spirometry gases, preoperative pulmonary comorbidities, smoking, surgical and anaesthetic strategies (6, 8, 9, 25–29), as well as various predictive risk models (30), research directly exploring how primary CRC sites influence lung injury and whether it correlates with the development of PPCs is limited. This study was designed meticulously to ascertain whether a causal correlation exists between these two variables, utilising propensity score matching to eradicate the bias introduced by other factors.

## 2 Materials and methods

### 2.1 Ethical compliance and clinical trial registry

This retrospective study was rigorously conducted at the Cancer Hospital, Chinese Academy of Medical Sciences (Beijing, China). The experimental protocol was thoroughly reviewed and approved by the institutional ethics committee (Approval No. 22/519-3721). Patients enrolled in the initial clinical trial provided informed written consent before commencing treatment. Both treatment protocols and the collation of clinical data were reserved for future scholarly analysis. Patient follow-up was maintained throughout the patients’ hospital stay and was sustained postdischarge via telecommunication and outpatient records. The inclusion criteria were as follows: (1) patients who underwent radical laparoscopic resection for colorectal cancer; (2) male and female patients aged between 18 and 80 years; (3) American Society of Anaesthesiologists (ASA) physical status ≤ III; (4) body mass index (BMI) < 40 kg/m²; (5) elective laparoscopic colorectal surgery that is expected to last for at least 2 h; and (6) intermediate or high risk of postoperative pulmonary complications (PPCs) as defined by the Assess Respiratory Risk in Surgical Patients in Catalonia (ARISCAT) score (a score of 26–44 is defined as intermediate risk and a score of > 44 is defined as high risk). The exclusion criteria were as follows: (1) aged < 18 years or > 80 years; (2) pregnant or breast-feeding status; (3) ASA physical status of IV or higher; (4) had a history of recent pulmonary infection/prior lung or abdominal surgery; (5) pneumothorax/giant bullae/chronic obstructive pulmonary disease; (6) severe cerebral and cardiovascular dysfunction/chronic renal failure with dialysis/hepatic dysfunction (Child−Pugh grade B or C)/immunologic or neuromuscular disease; (7) emergency or unplanned surgery; (8) expected prolonged mechanical ventilation after surgery; (9) allergies or contraindications to sevoflurane, propofol, or nonsteroidal anti-inflammatory drugs (NSAIDs); (10) participation in other drug trials within 1 preoperative month; and (11) distant metastasis.

### 2.2 Selection of participants

A total of 340 patients who underwent laparoscopic procedures at the National Cancer Center between April 2019 and May 2023 and were subsequently pathologically diagnosed with CRC were selected for this study. Comprehensive clinical information, including demographic and baseline data, intraoperative procedures, and follow-up outcomes, was recorded. The patient cohort was segmented into two separate categories according to the surgical position: the rectosigmoid colon group (RSC group), with the steep Trendelenburg position, and the descending/ascending colon group (DAC group), with a slightly inclined or reversed Trendelenburg position. Propensity score matching (PSM) was subsequently applied to achieve equilibrium in clinicopathological features.

### 2.3 Methodologies and interventions

Standard clinical practice guided the preoperative preparation and intraoperative monitoring. A lung-protective ventilation approach was utilised for all patients during anaesthesia, maintaining a tidal volume (TV) of 6–8 ml/kg predicted body weight (PBW) (31), accompanied by the same alveolar recruitment manoeuvres (ARMs, every 30 minutes) and positive end-expiratory pressure (PEEP=5 cmH_2_O).

### 2.4 Measurement of outcomes

PPCs were systematically categorised from 1–4 by the criteria delineated by Kroenke et al. (**Table 1**) (32). The clinical diagnosis of PPCs was substantiated by an independent radiologist utilising a routine low-dose computed tomography (CT) scan of the chest on postoperative day (POD) 3-4. The diagnosis and grading of PPCs were performed independently by a multidisciplinary team comprising medical oncologists, attending surgeons, and intensivists with senior clinical experience. Patients were assessed daily (9:00 to 10:00 a.m.) during the first postoperative week and subsequently weekly from the second to the fourth postoperative week. The plasma soluble forms of the receptor for advanced glycation end products (sRAGE), angiopoietin-2 (ANG-2) and interleukin-1β/6 [IL-1β/IL-6] levels were determined at three time points: preoperatively, on POD 1, and POD 3. In addition, systemic inflammatory markers (interleukin-6, interleukin-1β) and postoperative recovery in the two groups were also analysed for comparison. Furthermore, surgical rating scales (SRS) for the assessment of surgical operating conditions (5 score for optimal; 4 score for good; 3 score for acceptable; 2 score for poor; and 1 score for extremely poor) were administered by experienced surgeons (33), who were blinded to the PnP level to maintain interrater and intrarater agreement.

**Table 1:** Operational definitions of postoperative pulmonary complications ^a^.

Grade	Definition
1	Cough, dry Microatelectasis: abnormal lung findings and temperature **>** 37.5°C without other documented cause; results of chest radiograph either normal or unavailable Dyspnoea, not due to other documented cause
2	Cough, productive, not due to other documented cause Bronchospasm: new wheezing or pre-existent wheezing resulting in change therapy Hypoxemia: alveolar-arterial gradient **>** 29 and symptoms of dyspnoea or wheezing Atelectasis: radiological confirmation plus either temperature **>** 37.5 °C or abnormal lung findings Hypercarbia, transient, requiring treatment, such as naloxone or increased manual or mechanical ventilation Adverse reaction to pulmonary medication
3	Pleural effusion, resulting in thoracentesis Pneumonia, suspected: radiological evidence without bacteriological confirmation Pneumonia, proved: radiological evidence and documentation of pathological organism by Gram stain or culture Pneumothorax Reintubation postoperative or intubation, period of ventilator dependence (noninvasive or invasive ventilation) ≤ 48 h
4	Ventilatory failure: postoperative ventilator dependence ≥ 48 h, or reintubation with subsequent period of ventilator dependence ≥ 48 h

**^a^** Source: Kroenke et al. (32).

### 2.5 Statistical analyses

The process of PSM incorporated factors such as respiratory comorbidity (chronic obstructive pulmonary disease/chronic bronchitis vs. no relevant history), smoking status (either current/former smoker vs. nonsmoker), and age (younger than 60 years vs. 60 years or older), and a 1:2 matching ratio and a calliper of 0.05 were applied via the “MatchIt” R package. The absolute standardised difference (ASD) method, facilitated by the “stddiff” R package, was utilised to juxtapose the characteristics of the two groups and establish a balance, with an ASD value exceeding 0.2 indicating imbalance.

A logistic regression model was used to compare intraoperative procedures and follow-up outcomes between the two groups, and the results are expressed as odds ratios (ORs) and 95% CIs. Kaplan−Meier analysis, along with log-rank tests, was employed to assess the relationship between PPCs incidence and postoperative onset. Univariable or multivariable Cox proportional hazard models were applied to compare the incidence of PPCs within 30 days postoperatively, yielding hazard ratios (HRs) and 95% CIs. The overlap weight-based propensity score weighting method implemented via the R package “WeightIt” was used to eliminate bias by equalizing the distributions of covariates on the basis of the multivariable Cox proportional hazards model.

To determine the valid causal roles of variables of interest in the incidence of PPCs, a causal mediation analysis based on an accelerated failure time (AFT) survival regression model was performed using the R packages “mediation” and “survival”. The primary CRC site was designated as the exposure, the incidence and time of PPCs were the outcomes. A Poisson regression framework was employed to characterise the impact of exposure on postoperative gastrointestinal recovery time (mediator, ordered categorical variable), while age (binary, ≥60 years old and <60 years old) and anaesthetic duration (binary, more than and less than 3 hours), which are associated with PPCs incidence in the univariable Cox proportional hazards model, were included in the analysis as covariates. Additionally, a logistic regression approach was used to analyse the effects of exposure on age (mediator) when postoperative gastrointestinal recovery time and anaesthetic duration were included as covariates. Furthermore, the causal mediation analysis was adjusted for the following baseline and clinical confounders related to the primary CRC site: TNM stage (categorical, Tis-stage I, stage II, and stage III-IV), history of neoadjuvant therapy (binary, yes and no), preoperative haemoglobin levels (continuous), blood product transfusion (binary, yes and no) and duration of surgery (continuous). The average causal mediation effects (ACME) were calculated to measure the mediating impact of gastrointestinal recovery time or age on PPCs incidence. The average direct effects (ADE) were the estimation of exposure on the outcome that mediators cannot explain. The results of the AFT models are described in terms of acceleration regression coefficients, in which a negative coefficient indicates a shorter occurrence time of PPCs.

Quantitative variables were analysed with Student’s t test or the Mann−Whitney U test, whereas categorical data were evaluated via the chi−square test or Fisher’s exact test. A two-sided P value < 0.05 was considered statistically significant. Data analyses were performed using SPSS 25 (IBM SPSS Inc., USA) or R software.

## 3 Results

### 3.1 Characteristics and treatment of the patients

Three hundred forty CRC patients matched the criteria for inclusion in the study database. Following PSM, 300 patients were divided into two groups, comprising 200 patients in the RSC group and 100 patients in the DAC group. The median ages were 63.0 and 61.0 years for the two groups. Most characteristics at baseline were comparable across the two groups, with the exception of a greater prevalence of tumour stage II - IV (RSC vs. DAC; 75.0% [150 of 200] vs. 85.0% [85 of 100], ASD=0.253) and a subsequent lower proportion of neoadjuvant therapy (RSC vs. DAC; 14.5% [29 of 200] vs. 5.0% [5 of 100], ASD=0.324), lower haemoglobin levels (g/L; RSC vs. DAC; 137.5 [127.0-152.8] vs. 130.0 [107.5-144.8], ASD=0.570), an enhanced proportion of anaemia (RSC vs. DAC; 21.0% [42 of 200] vs. 41% [41 of 100], ASD=0.443) and more blood product transfusions (RSC vs. DAC; 5.0% [10 of 200] vs. 15.0% [15 of 100], P=0.003) in the DAC group than in the RSC group (**Tables 2**, **3**). Moreover, the DAC group required extended anaesthetic (min; median [interquartile range, IQR]; RSC vs. DAC; 170.0 (145.0–207.8) vs. 199.5 (162.5–221.8), P<0.001) and surgical duration (RSC vs. DAC; 145.0 (112.0–180.0) vs. 165.0 (132.5–193.0), P=0.002) (**Table 3**). In a predefined subgroup analysis, there was a notable increase in the duration of anaesthesia from the sigmoid (median, 160.0 min; IQR, 127.3-192.0 min), rectal (median, 170.0 min; IQR, 150.0-225.0 min), and ascending (median, 190.5 min; IQR, 160.0-216.3 min) to the descending colon (median, 212.0 min; IQR, 174.5-235.3 min). Similarly, the surgical duration showed a trend comparable to that of the sigmoid (median, 126.5 min; IQR, 100.0–152.5 min), rectal (median, 150.0 min; IQR, 131.0–200.0 min), ascending (median, 163.5 min; IQR, 131.5–187.0 min), and descending colon (median, 175.0 min; IQR, 149.3–208.8 min).

**Table 2:** Demographic and baseline data.

Characteristic	Rectosigmoid colon Group (RSC, N=200)	De/Ascending colon Group (DAC, N=100)	ASD
Age, yr	63.0 (54.0-68.0)	61.0 (52.0-68.8)	0.083
Gender, Male	123 (61.5%)	62 (62.0%)	0.010
Body mass index (BMI), kg/m^2^	24.7 ± 3.5	24.9 ± 3.5	0.062
Obesity (Chinese standard*)	33 (16.5%)	22 (22.0%)	0.140
Alcoholism**	33 (16.5%)	16 (16.0%)	0.014
Allergic history	21 (10.5%)	10 (10.0%)	0.016
Smoking status			0.057
Nonsmoker	142 (71.0%)	71 (71.0%)	
Former smoker#	33 (16.5%)	18 (18.0%)	
Current smoker	25 (12.5%)	11 (11.0%)	
Dry cough/Expectoration	21 (10.5%)	8 (8.1%)	0.083
Respiratory comorbidity			0.177
No relevant history	150 (75%)	75 (75%)	
COPD/Chronic bronchitis	47 (23.5%)	25 (25.0%)	
Asthma	3 (1.5%)	0 (0.0%)	
Hypertension	86 (43.0%)	45 (45.0%)	0.040
Coronary heart disease	14 (7.0%)	7 (7.0%)	<0.001
Cerebrovascular disease	14 (7.0%)	4 (4.0%)	0.132
Diabetes mellitus	37 (18.5%)	12 (12.0%)	0.182
ASA status			0.057
I	8 (4.0%)	3 (3.0%)	
II	179 (89.5%)	90 (90.0%)	
III	13 (6.5%)	7 (7.0%)	
TNM staging†			0.253
Tis -Stage I	50 (25.0%)	15 (15.0%)	
Stage II	65 (32.5%)	36 (36.0%)	
Stage III- IV	85 (42.5%)	49 (49.0%)	
Adjuvant Chemo/Radiotherapy	29 (14.5%)	5 (5.0%)	0.324
Anaemia††	42 (21.0%)	41 (41.0%)	0.443
Preoperative routine blood test			
Haemoglobin, g/L	137.5 (127.0-152.8)	130.0 (107.5-144.8)	0.570
White blood cell, 10^9^/L	6.0 (5.1-7.1)	6.3 (5.0-7.5)	0.121
Neutrophils, 10^9^/L	3.8 (3.0-4.6)	3.8 (2.9-5.0)	0.060

All data are presented as the means ± SDs, n (%), or medians (interquartile ranges). An imbalance between two groups was defined as an absolute standardised difference (ASD) value greater than 0.200. *Chinese standard for obesity: underweight (< 18.5 kg/m^2^), normal weight (18.5-23.9 kg/m^2^), overweight (24.0-27.9 kg/m^2^), or obese (≥ 28.0 kg/m^2^). **Alcoholism: daily consumption of the equivalent of 80 g of alcohol for at least 5 years. # Former smoker: weaning more than 4 weeks. †TNM staging was defined by the NCCN guidelines (Colon/Rectal Cancer) Version 1.2022 (www.nccn.org/patients). ††Anaemia was defined as haemoglobin levels of < 13.0 g/dL and 12.0 g/dL for males and females, respectively.

BMI, body mass index; COPD, chronic obstructive pulmonary disease; ASA, American Society of Anaesthesiologists; Tis, tumour *in situ*.

**Table 3:** Intraoperative procedures.

	Rectosigmoid colon Group (RSC, N=200)	De/Ascending colon Group (DAC, N=100)	*P* value
Intraoperative ventilation index			
Tidal volume (ml/kg of predicted body weight)	6-8	6-8	—
Inspired oxygen fraction, (FiO_2_, %)	40-60	40-60	—
Intraoperative PEEP (cmH_2_O)	5	5	—
Anaesthetic strategy (%)			0.695
Inhalation	177 (88.5)	90 (90.0)	
TIVA	23 (11.5)	10 (10.0)	
Medication			
Propofol (Intubation, mg)	140.0 (120.0-180.0)	150.0 (120.0-180.0)	0.081
Sufentanil (ug)	35.0 (35.0-40.0)	35.0 (35.0-40.0)	0.907
Remifentanil (ug)	960.0 (700.0-1225.0)	850.0 (642.5-1150.0)	0.967
Rocuronium (Intubation, mg)	50.0 (40.0-50.0)	50.0 (45.0-50.0)	0.347
Rocuronium (Maintenance, mg)	40.0 (20.0-60.0)	30.0 (20.0-50.0)	0.593
Duration (min)			
Anaesthesia	170.0 (145.0-207.8)	199.5 (162.5-221.8)	<0.001
Surgery	145.0 (112.0-180.0)	165.0 (132.5-193.0)	0.002
Category of anaesthetic duration (%)			<0.001
≤ 3 hours	130 (65.0)	35 (35.0)	
> 3 hours	70 (35.0)	65 (65.0)	
Assessment of surgical condition			
SRS Score	5 (5-5)	5 (4-5)	0.003
Volume of fluids administered (ml)			
Crystalloid	1100.0 (1000.0-1500.0)	1100.0 (1000.0-1237.5)	0.192
Colloid	500.0 (0-500.0)	500.0 (500.0-500.0)	0.161
Blood products transfusion (%)	10 (5.0)	15 (15.0)	0.003
Estimated blood loss (ml)	37.5 (20.0-80.0)	50.0 (20.0-100.0)	0.254
Urine volume (ml)	300.0 (200.0-300.0)	300.0 (300.0-300.0)	0.185

All data are presented as the means ± SDs, n (%), or medians (interquartile ranges). FiO_2_, fraction of inspired oxygen; PEEP, positive end expiratory pressure; TIVA, total intravenous anaesthesia; SRS, surgical rating scale.

In addition, the Mann−Whitney rank test revealed that the surgical conditions, as evaluated by the surgical rating scale (SRS), were poorer in the DAC group than in the RSC group (RSC vs. DAC; 5 [5.0–5.0] vs. 5.0 [4.0–5.0]; P=0.003, **Table 3**). Details of the mechanical parameters of pulmonary compliance and peak and plateau airway pressures at different surgical positions at the tumour sites are provided in **Table 4**.

**Table 4:** Mechanical ventilation parameters for different anaesthetic methods.

Characteristic	Rectosigmoid colon Group (RSC, N=200)	De/Ascending colon Group (DAC, N=100)		*P* value*	*P* value#
	25-30° Trendelenburg	10-15° Reverse Trendelenburg	10-15°Trendelenburg		
Ppeak, cmH_2_O	27.0 (24.0-32.0)	22.0 (21.0-23.5)	25.0 (24.0-28.5)	<0.001	0.120
Pplate, cmH_2_O	23.0 (21.0-27.0)	18.0 (17.0-20.0)	23.0 (21.5-26.5)	<0.001	0.391
Respiratory rate, min	14.0 (13.0-16.0)	13.0 (12.0-15.0)	13.0 (12.0-16.0)	0.006	0.029
EtCO_2_, mmHg	39.0 (36.0-42.0)	30.0 (28.0-33.0)	32.0 (29.0-34.0)	<0.001	<0.001
Dynamic C_RS_, ml/cmH_2_O	21.1 (20.4-21.6)	26.7 (24.7-29.1)	22.6 (21.3-26.0)	<0.001	<0.001
Static C_RS_, ml/cmH_2_O	25.5 (24.4-26.5)	34.2 (31.6-37.6)	25.3 (23.3-27.8)	<0.001	0.817

The data are shown as n (%) or medians (interquartile ranges).

The quantitative data were compared via the Mann−Whitney rank test.

*Comparison between the Trendelenburg position (25–30°) and the reverse Trendelenburg position (10–15°). # Comparison between the Trendelenburg position (25–30°) and the Trendelenburg position (10–15°).

Ppeak, peak airway pressure; Pplat, plateau pressure; EtCO2, end-tidal carbon dioxide; C_RS_, respiratory system compliance.

### 3.2 Outcomes with respect to efficacy

First, among the indicators related to postoperative recovery, there was no significant difference in postanaesthesia care unit (PACU) recovery time or postoperative hospitalisation days between the DAC and RSC groups. However, a greater protracted gastrointestinal recovery time (defined as the period of first flatus or stool through the anus after surgery) was observed in the DAC group (DAC vs. RSC; 3.0 [2.0–3.0] vs. 3.0 [2.0–3.0]; OR=1.698; 95% CI, 1.098 to 2.684; P=0.020; **Table 5**). Moreover, the gastrointestinal recovery time was longer in patients with PPCs than in those without (with PPCs vs. without PPCs; 3.0 [2.0–3.0] vs. 2.5 [2.0–3.0]; OR=2.114; 95% CI, 1.361 to 3.362; P=0.001).

**Table 5:** Effectiveness outcomes.

	Rectosigmoid colon Group (RSC, N=200)	De/Ascending colon Group (DAC, N=100)	OR (95% CI)	*P* value*
Postoperative recovery				
PACU time, min	20.0 (15.0-30.0)	20.0 (15.0-29.5)	0.996 (0.978-1.014)	0.700
Gastrointestinal recovery time, days	3.0 (2.0-3.0)	3.0 (2.0-3.0)	1.698 (1.098-2.684)	0.020
Postoperative hospitalisation, day	6.0 (6.0-8.0)	7.0 (6.0-8.0)	1.044 (0.934-1.165)	0.439
Mechanical lung injury				
sRAGE, pg/ml				
Preoperation	497.4 (329.7-862.2)	474.5 (348.2-659.3)	1.000 (0.998-1.001)	0.452
POD 1	1271.6 (896.3-1587.6)	1029.6 (576.8-1365.2)	0.999 (0.998-1.000)	0.007
POD 3	1063.3 (618.4-1335.4)	881.1 (510.4-1060.4)	0.999 (0.998-1.000)	0.077
ANG-2, ng/ml				
Preoperation	4.3 (2.3-7.4)	3.6 (2.1-8.1)	1.050 (0.981-1.127)	0.155
POD 1	11.3 (7.6-15.3)	9.9 (4.7-13.4)	0.993 (0.928-1.054)	0.816
POD 3	8.7 (5.6-12.3)	8.2 (4.3-10.9)	1.009 (0.939-1.080)	0.792
Systemic inflammatory index				
IL-1β, pg/ml				
Preoperation	2.9 (2.4-3.3)	2.6 (2.4-3.1)	0.765 (0.470-1.048)	0.170
POD 1	3.7 (3.0-5.0)	4.4 (3.1-6.4)	1.024 (0.983-1.076)	0.249
POD 3	3.1 (2.6-3.6)	3.2 (2.7-4.0)	0.990 (0.905-1.039)	0.740
IL-6, pg/ml				
Preoperation	2.1 (1.6-2.3)	2.1 (1.6-2.3)	0.820 (0.348-1.803)	0.633
POD 1	2.5 (2.1-3.0)	2.6 (2.3-4.4)	1.073 (0.977-1.214)	0.171
POD 3	2.2 (1.7-2.4)	2.2 (1.7-2.5)	1.155 (0.755-1.735)	0.478
Incidence of PPCs within 30 days, cases (%)	60 (30.0)	48 (48.0)	1.669 (1.141-2.439)	0.008^#^
Kroenke grade				
1	43 (21.5)	23 (23.0)	1.111 (0.669-1.844)	0.684^#^
2-3	17 (8.5)	25 (25.0)	0.875 (0.465-1.647)	0.679^#^
Radiographic diagnosis				
Microatelectasis	41 (20.5)	24 (24.0)	1.211 (0.732-2.005)	0.456^#^
Atelectasis	10 (5.0)	17 (17.0)	3.526 (1.614-7.703)	0.002^#^
Pleural Effusion	12 (6.0)	16 (16.0)	2.787 (1.318-5.895)	0.007^#^
Pneumonia	7 (3.5)	7 (7.0)	2.171 (0.761-6.197)	0.147^#^

The data are shown as n (%) or medians (interquartile ranges).

*Except specifically note that the odds ratios (95% CI) and their relative *P* values were calculated by the logistic regression model.

^#^Hazard ratios (95% CIs) and their relative *P* values were calculated by the univariate Cox proportional hazards model.

sRAGE, soluble form of the receptor for advanced glycation end products; ANG-2, angiopoietin-2; PPCs, postoperative pulmonary complications; IL-1β, interleukin-1β; IL-6, interleukin-6; PACU, postanaesthesia care unit; POD, postoperative day.

Next, the plasma levels of sRAGE on POD1 were greater in the patients in the RSC group than in those in the DAC group (DAC vs. RSC; 1029.6 [576.8–1365.2] vs. 1271.6 [896.3–1587.6]; OR=0.999; 95% CI, 0.998 to 1.000; P=0.007), which also indicated marginal significance for the two groups on POD3 (DAC vs. RSC; 881.1 [510.4–1060.4] vs. 1063.3 [618.4–1335.4]; OR=0.999; 95% CI, 0.998 to 1.000; P=0.077; **Table 5**). However, the levels of the endothelial injury indicator ANG-2 and the systemic inflammatory indicators interleukin-1β (IL-1β)/IL-6 remained unchanged across the two cohorts (P>0.05, **Table 5**). In addition, no significant relationship was detected between the plasma biomarkers sRAGE, ANG-2, IL-1β, or IL-6 and the PPCs incidence (P>0.05).

**Figure 1:**
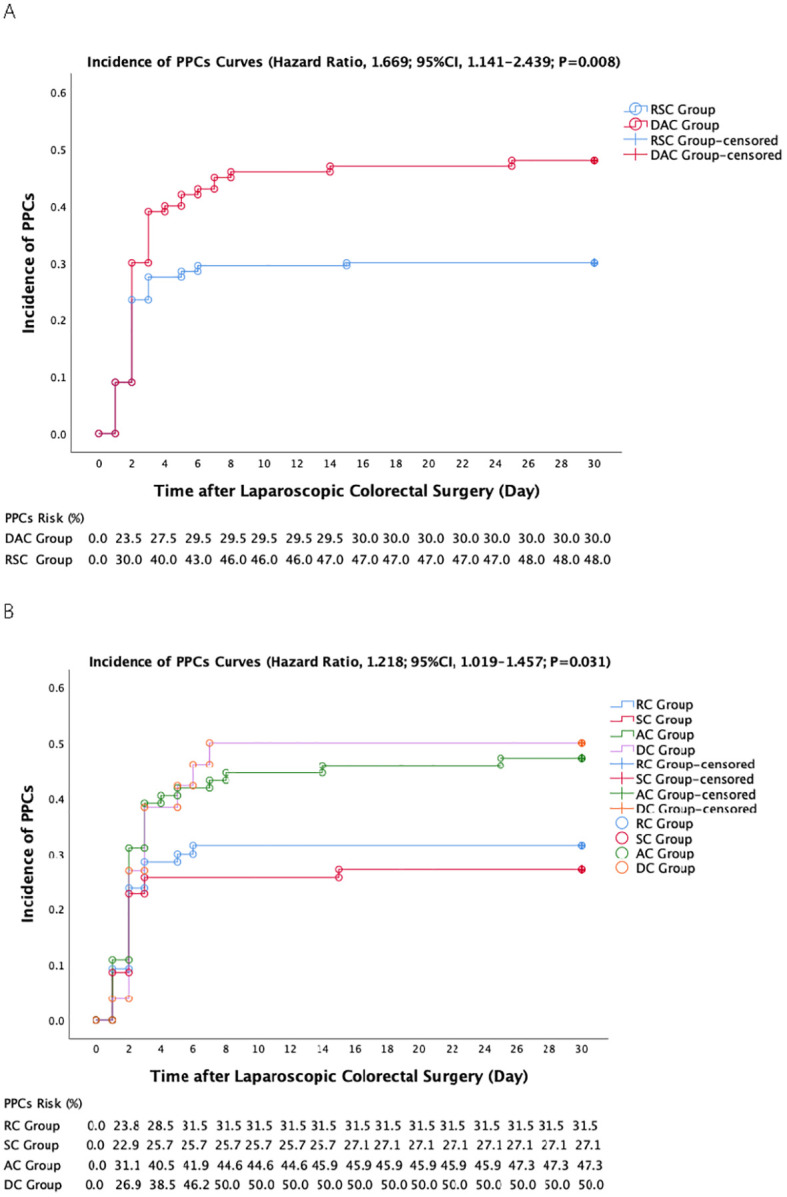
Curves representing the incidence of postoperative time-to-PPCs within a 30-day postoperative period (univariate Cox proportional hazards regression analysis). **(A)** Incidence of PPCs in the descending/ascending colon (DAC) group and the rectosigmoid colon (RSC) group. **(B)** Incidence of PPCs in subgroups of various colorectal positions. DC, descending colon; AC, ascending colon; SC, sigmoid colon; RC, rectum of the colon.

Furthermore, an unexpected finding was that the DAC group exhibited a significantly greater occurrence of PPCs within a span of 30 days postsurgery. The hazard ratio (HR) was 1.669 (95% CI, 1.141 to 2.439; P=0.008; **Table 5**), with a 48% incidence (48/100) in the DAC group and a 30.0% incidence (60/200) in the RSC group (**Figure 1A**). Moreover, the DAC group also had a significantly greater incidence of atelectasis (P=0.002) and pleural effusion (P=0.007; **Table 5**). Notably, in a preplanned subgroup analysis, a significant increase in the incidence of PPCs in the sigmoid colon, rectum, ascending colon, and descending colon was detected (HR=1.218; 95% CI, 1.019 to 1.457; P=0.031; **Figure 1B**). Among the observed incidents, microatelectasis was the most prevalent, occurring in 21.7% (65 out of 300) of the cases.

### 3.3 Tumour occurrence in the descending/ascending colon was an independent risk factor for PPCs outcomes

The univariable Cox proportional hazards model identified four PPCs-related factors with statistical significance. These factors included an anaesthetic duration exceeding 3 hours (HR=1.508; 95% CI, 1.033 to 2.202; P=0.033), an age of 60 years or more (HR=1.708; 95% CI, 1.126 to 2.593; P=0.012), a primary CRC site in the DAC (HR=1.669; 95% CI, 1.141 to 2.439; P=0.008) and a gastrointestinal recovery time (HR=1.775; 95% CI, 1.219 to 2.584; P=0.003). The time-to-PPCs were subsequently analysed using these high-risk factors through a multivariable Cox proportional hazards regression model. The findings revealed that gastrointestinal recovery time (HR=1.750; 95% CI, 1.198 to 2.555; P=0.004), age (60 years and above vs. under 60 years, HR=1.678; 95% CI, 1.103 to 2.552; P=0.016) and primary CRC site (DAC vs. RSC; HR=1.470; 95% CI, 0.9847 to 2.194; P=0.059) were significant or marginal independent risk factors for PPCs incidence. To acquire unbiased estimates of the influence of these factors on PPCs, the adjusted HRs of the primary CRC site, age and gastrointestinal recovery time were 1.634 (95% CI, 1.115 to 2.396; P=0.012), 1.745 (95% CI, 1.131 to 2.692; P=0.012) and 1.750 (95% CI, 1.159 to 2.644; P=0.008), respectively, according to propensity score weighting via overlap weights. In summary, even when adjustments were made in populations with significant differences in other covariates, such as age and gastrointestinal recovery time, the primary CRC site emerged as a stand-alone risk factor for PPCs.

### 3.4 The impact of the primary CRC site in the DAC on PPCs occurrence cannot be attributed to the other established clinical and baseline variables

**Figure 2:**
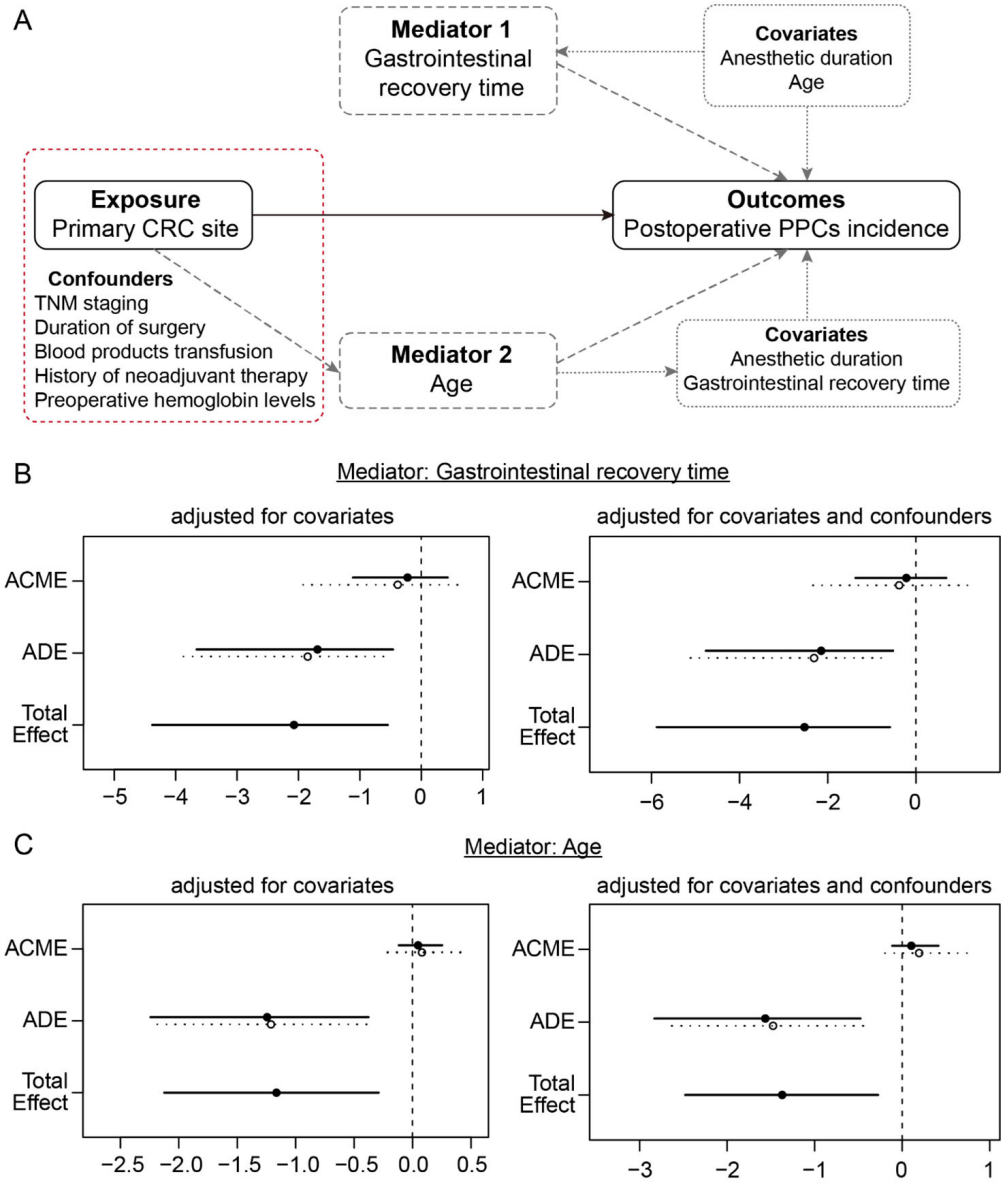
Causal mediation analysis of the primary CRC site (exposure), gastrointestinal recovery time (mediator 1), age (mediator 2) and incidence of PPCs (outcome). **(A)** Directed acyclic graph of the mediation model. The measured covariates are included as fixed effects in each model, in the absence or presence of the confounders that are associated with the primary CRC site. **(B)** Mediation analysis of the models with mediator 1 (gastrointestinal recovery time) in the absence or presence of confounders. **(C)** Mediation analysis of the models with mediator 2 (age) in the absence or presence of confounders.

The aforementioned analyses demonstrated that the primary CRC site was associated with several baseline and clinical characteristics and PPCs incidence. To address whether the primary CRC site is impacted by other clinical characteristics and, in turn, influences the incidence of PPCs, causal mediation analysis using the AFT survival regression model with the quasi-Bayesian Monte Carlo method was performed (**Figure 2A**). First, when the gastrointestinal recovery time was designated as a mediator, the primary CRC site had a significant average direct effect (ADE; effect=-1.769 [95% CI, -3.742 to -0.49]; P=0.004) and total effect (effect=-2.072 [95% CI, -4.380 to 0.54]; P=0.004) on increasing the incidence of PPCs, whereas the average causal mediation effect (ACME) was not significant (effect=-0.303 [95% CI, -1.512 to 0.58]; P=0.474; **Figure 2B**). Even after adjusting for the other confounders associated with the primary CRC site, the mediation effect estimates did not significantly change (**Figure 2B**). When age was considered a mediator, the ACME, ADE, and total effects were 0.063 (95% CI, -0.164 to 0.34; P=0.596), -1.228 (95% CI, -2.218 to -0.37; P=0.002) and -1.165 (95% CI, -2.125 to -0.29; P=0.004), respectively (**Figure 2C**). Similarly, the tendency was not significantly altered after adjusting for confounders (**Figure 2C**). Therefore, compared with those in the RSC group, the occurrence of tumours in the descending/ascending colon (DAC group) directly resulted in an increased incidence of PPCs, and other clinical characteristics, such as the gastrointestinal recovery time and age of patients, did not mediate this effect.

## 4 Discussion

In the laparoscopic procedure for CRC, anterior resection of rectal cancer and sigmoidectomy necessitate the sustained steep Trendelenburg position, where the patient’s head is positioned lower than the feet at an angle of approximately 25–30°. In contrast, ascending and descending colectomies require intermittent transformations of the Trendelenburg position of ±10–15°. Research has indicated that rapid atelectasis involvement can develop in lung areas with high pressure within 10 minutes in the Trendelenburg position (23). The significant discrepancy in peak and plateau airway pressures and pulmonary compliance between these two surgical positions (**Table 4**) may precipitate various pulmonary stresses and lung injuries (21–23, 29). This process is further complicated by carbon dioxide pneumoperitoneum, which exacerbates atelectasis, inflammation and lung injury via hypercapnia and respiratory acidosis. Consequently, the airway pressure (barotrauma) resulting from laparoscopy may surpass the elastic limits of the lung parenchyma, generating transpulmonary pressures that damage the alveolar epithelium (34, 35). Our results in **Table 4** show that patients in the Trendelenburg position (25–30°) had higher peak airway pressure (Ppeak) and plateau airway pressure (Pplat) and lower pulmonary compliance than patients in the reverse Trendelenburg position did (P<0.001, **Table 4**). The abdominal contents move upwards towards the diaphragm in the Trendelenburg position, increasing intrathoracic cavity pressure and airway pressure and decreasing lung compliance. Once a threshold is overcome, excessive airway pressure and lung strain deform epithelial and endothelial cells (known as barotrauma), resulting in secondary stress fatigue, microcapillary rupture and inflammation (35, 36). Inflammation triggered by damage can be perpetuated by the activation of various signalling pathways, resulting in further biotrauma to the lung. Hence, patients with worse dynamic ventilation parameters can suffer worse lung injuries.

To characterise the magnitude of intraoperative general and pulmonary inflammation and lung injury, our research applied several biological biomarkers. Receptors for advanced glycation end products (RAGE), especially sRAGE, are widely studied as indicators of pulmonary injury because of their high expression in type I pneumocytes (37–39). To the best of our knowledge, plasma sRAGE is the only biomarker that has been correlated with a functional readout of epithelial barrier function and alveolar fluid clearance (40, 41). sRAGE has been extensively implicated in the process of inflammation and lung injury to alveolar epithelial cells (42, 43), particularly in the context of mechanical high tidal volume-induced alveolar overdistension or acute respiratory distress syndrome (ARDS) (44, 45). Previous study demonstrated that sRAGE is strongly linked to the duration of ventilation for POD 1, with a 25% multiplicative increase in hours of ventilation (95% CI, 2% to 52%, P = 0.03) observed for every 50 pg/ml increase in sRAGE (46). In addition to sRAGE, another major form of RAGE is anchored to the cell membrane (mRAGE) (39, 47). mRAGE is capable of binding proinflammatory ligands in the immune microenvironment, such as AGEs, S100 proteins and high mobility group box 1 (HMGB1), and consequently activates downstream inflammatory pathways, such as nuclear factor kappa-light-chain enhancer of activated B cells (NF-κB) and signal transducer and activator of transcription 3 (STAT3). Stimulation of these pathways can lead to cytokine release, cell migration and upregulated RAGE expression, which in turn perpetuates the inflammatory cascade, resulting in the occurrence and progression of many pathological processes and diseases, including airway inflammation and acute lung injury (ALI) (48). Moreover, the extracellular part of mRAGE is cleaved from the membrane upon lung injury and becomes sRAGE, which can be detected in blood and bronchial lavage fluid. sRAGE functions as a “decoy”, as it attaches to ligands of RAGE without activating intracellular pathways and therefore prevents excessive cell injury and airway inflammation by decreasing the expression and release of downstream cytokines, such as tumour necrosis factor-alpha (TNF-α), IL-1β and IL-6 (47, 49). The interactions between RAGE and certain ligands and sequential pathways constitute the RAGE axis. Since the RAGE axis effectively modulates inflammation, several benign and malignant diseases that are strongly associated with the immune microenvironment can be affected by the function of RAGE (50). The extant literature provides substantial support for the proposition that endogenous plasma sRAGE plays a mechanistic role in the pathophysiology of ARDS in models of lung injury that involve acid and hyperoxia (37, 51, 52). Additionally, existing studies further corroborate the pivotal function of endogenous plasma sRAGE within the AGE-RAGE axis as a prospective catalyst of lung injury, indicating that there is potential for reducing plasma sRAGE levels or targeting the AGER/sRAGE axis to have a preventative or therapeutic effect on inflammation-related lung disease (53–55). Notably, sRAGE is also regarded as a promising biomarker for colon carcinoma. Aglago et al. reported an inverse association between prediagnostic sRAGE levels and colorectal risk in men (56).

ANG-2 is another potential candidate for endothelial inflammation and injury caused primarily by ARDS and sepsis (57, 58). ANG-2 originates primarily from endothelial cells and acts as a key factor in the modulation of vascular permeability as an antagonist against ANG-1, which promotes endothelial permeability by preserving cellular junctions and signalling the reduction in certain surface-adhesion molecules through tyrosine kinases with immunoglobulin-like and EGF-like domain 2 (Tie-2) signalling pathways (59). As the highest Tie-2 expression in adult tissues is observed in the lung, disruption of the pathway caused by ANG-2 can lead to pulmonary disorders (60). Environmental factors related to pathogenesis, such as hypoxia and increased levels of cytokines, can lead to the production and release of ANG-2 (61), and existing studies have shown that ANG-2 is an essential biomarker of ALI/ARDS (62, 63). Moreover, ANG-2 has been shown to enhance tumour metastasis and immunosuppression by regulating cytokine levels in the tumour microenvironment, suggesting that ANG-2 is a potential target for cancer treatment therapy (64, 65). ANG-2 and vascular endothelial growth factor (VEGF) may promote the formation of new blood vessels through a synergistic effect, leading to liver metastasis of colorectal cancer (66). ANG-2-targeted comprehensive treatment has sparked new ideas in cancer treatment (67, 68).

Furthermore, IL-1β and IL-6 are major proinflammatory cytokines released by diverse immune and nonimmune cells upon stimulation, such as infection, injury, and stress. IL-1β originates primarily from macrophages, dendritic cells, and neutrophils, although it can also be secreted by epithelial cells and fibroblasts. Canonically, it is synthesised as an inactive precursor, which is cleaved and activated by caspase-1 upon environmental stimulation (69). However, the production of IL-6 is initiated by pathogen-associated molecular patterns (PAMPs) and damage-associated molecular patterns (DAMPs) (70). Various types of cells, including T cells, macrophages, and fibroblasts, are involved in the synthesis and secretion of IL-6. Both cytokines are essential for adjusting the immune microenvironment by triggering the activation of various immune cells, stimulating the differentiation of T helper cells, and contributing to the synthesis and secretion of acute-phase reactants in the liver. Owing to their pivotal roles in inflammation, IL-1β and IL-6 have been proposed as biomarkers of lung injury and airway inflammation, particularly in diseases such as chronic obstructive pulmonary disease (COPD), ALI and ARDS (62). In addition, during general anaesthesia, the expression and release of these cytokines can be stimulated by lung impairment caused by mechanical ventilation and the activation of pathways related to RAGE (47). The presence of these cytokines serves as indicators of ongoing immune activation and the extent of airway remodelling, making them useful for monitoring the magnitude and progression of lung injuries.

Inflammation has been demonstrated to play a critical role in the pathogenesis of PPCs following noncardiac surgery (46). Nevertheless, no correlation between the above inflammatory indicators and the incidence of PPCs was found by the analysis of the univariate Cox proportional hazards model in this study. This result is similar to those of many investigations. Aaron et al. identified sRAGE as a potential indicator of acute lung injury, with no significant correlation with PPCs (46); Neto et al. identified none of the plasma biomarkers expressed for inflammation or lung injury (TNF-α, IL-6 and IL-8; sRAGE, surfactant protein [SP]-D, Clara cell protein [CC]-16 and Krebs von den Lungen 6 [KL6]) is related to PPCs (71); Jabaudon et al. compared the sRAGE levels between patients who received protective ventilation and those who did not and concluded that patients with at least one hypoxemia episode presented elevated sRAGE levels during mechanical ventilation, but no association was observed between intraoperative changes in sRAGE and PPCs within 30 days postsurgery (72). The ambiguity of the correlation between plasma sRAGE and PPCs may be ascribed to the inflammatory consumption of sRAGE, as it works by attaching to ligands. Recent studies have demonstrated that postoperative sRAGE rapidly decreases to or below preoperative levels within 24 hours following cardiac surgery. Consequently, it is plausible that by the time samples were collected on POD 1, sRAGE values may have already returned to normal (46). Additionally, as the heterogeneity of patients and definitions of PPCs vary, not all lung injuries ultimately lead to PPCs.

Notably, patients who undergo proctosigmoidectomy (RSC group) with more pulmonary stress and injury are theoretically more susceptible to PPCs incidence. Our findings revealed a consistent correlation between the RSC group with the steep Trendelenburg position and lung epithelium injury, with higher sRAGE levels in the RSC group on the postoperative day (POD, P=0.007 for POD 1 and P=0.077 for POD 3 [marginal significance]; **Table 5**). This may be attributed to the greater degree of lung injury in patients in the RSC group suffered from steeper Trendelenburg position than that in the counterpart, as we expected. However, contrary to expectations, univariate and multivariate Cox regression analyses revealed a negative correlation between the primary CRC site and PPCs, with a significantly decreased incidence of PPCs in the RSC group (DAC vs. RSC; HR=1.669; 95% CI, 1.141 to 2.439; P=0.008). Our propensity score weighting method using overlap weights in a Cox proportional hazards regression analysis demonstrated that tumours occurring in the descending/ascending colon (DAC group) indeed increased the risk of PPCs (adjusted HR=1.634; 95% CI, 1.115 to 2.396; P=0.012). The subsequent causal mediation analysis demonstrated that this factor remains a direct effect on the incidence of PPCs when adjusted for several baseline confounders and clinical covariates related to the primary CRC site and PPCs.

The causes of DAC for the high incidence of PPCs are worthy of attention. Postoperative gastrointestinal recovery time, age and anaesthetic duration exceeding 3 hours were also unfavourable factors for the incidence of PPCs according to the univariate Cox proportional hazards model. It is well known that enhanced recovery is essential for reducing postoperative complications (73), especially for PPCs (28, 74). The literature indicates that right hemicolectomy is associated with inferior intestinal function and an elevated prevalence of metabolic syndrome due to factors such as bile acid malabsorption, impaired water reabsorption, small intestinal bacterial overgrowth, and gut microbial dysbiosis, which potentially deteriorates gastrointestinal recovery and facilitates inflammation and PPCs (75–77). In the present study, a greater gastrointestinal recovery time was indeed observed in the DAC group (P=0.020, **Table 5**), and patients with PPCs exhibited longer gastrointestinal recovery times (patients with PPCs vs. those without PPCs; 3.0 [2.0–3.0] vs. 2.5 [2.0–3.0]; OR=2.114; 95% CI, 1.361 to 3.362; P=0.001). Additionally, age serves as a robust marker for PPCs, even in healthy older patients at elevated risk of PPCs (8, 28). Previous studies have indicated that age over 60 years or 65 years is a risk factor for PPCs development (8, 78). Stratification analysis suggested that the risk of PPCs significantly increased with age, which may be related to the frailty of elderly patients with weakened respiratory muscle function (10, 27, 79). In terms of duration, the literature substantiates that a surgical or anaesthesia duration surpassing two hours independently contributes to PPCs, revealing a significant association between operative duration and PPCs. This association has odds ratios (and confidence intervals) of 4.9 (2.4 to 10.1) and 9.7 (4.7 to 19.9) for operative periods exceeding 2 and 3 hours, respectively (9, 27). Here, we identified an obvious correlation between primary tumour location and surgical/anaesthesia duration, as shown in **Table 3** (P<0.001 for anaesthetic duration, P=0.002 for surgical duration). For subgroup analysis, the median anaesthetic durations for the sigmoid (160.0 min), rectal (170.0 min), ascending (190.5 min) and descending (212.0 min) colon regions also showed apparent relevance to the corresponding time-to-PPCs incidence (P=0.031, **Figure 1B**).

**Figure 3:**
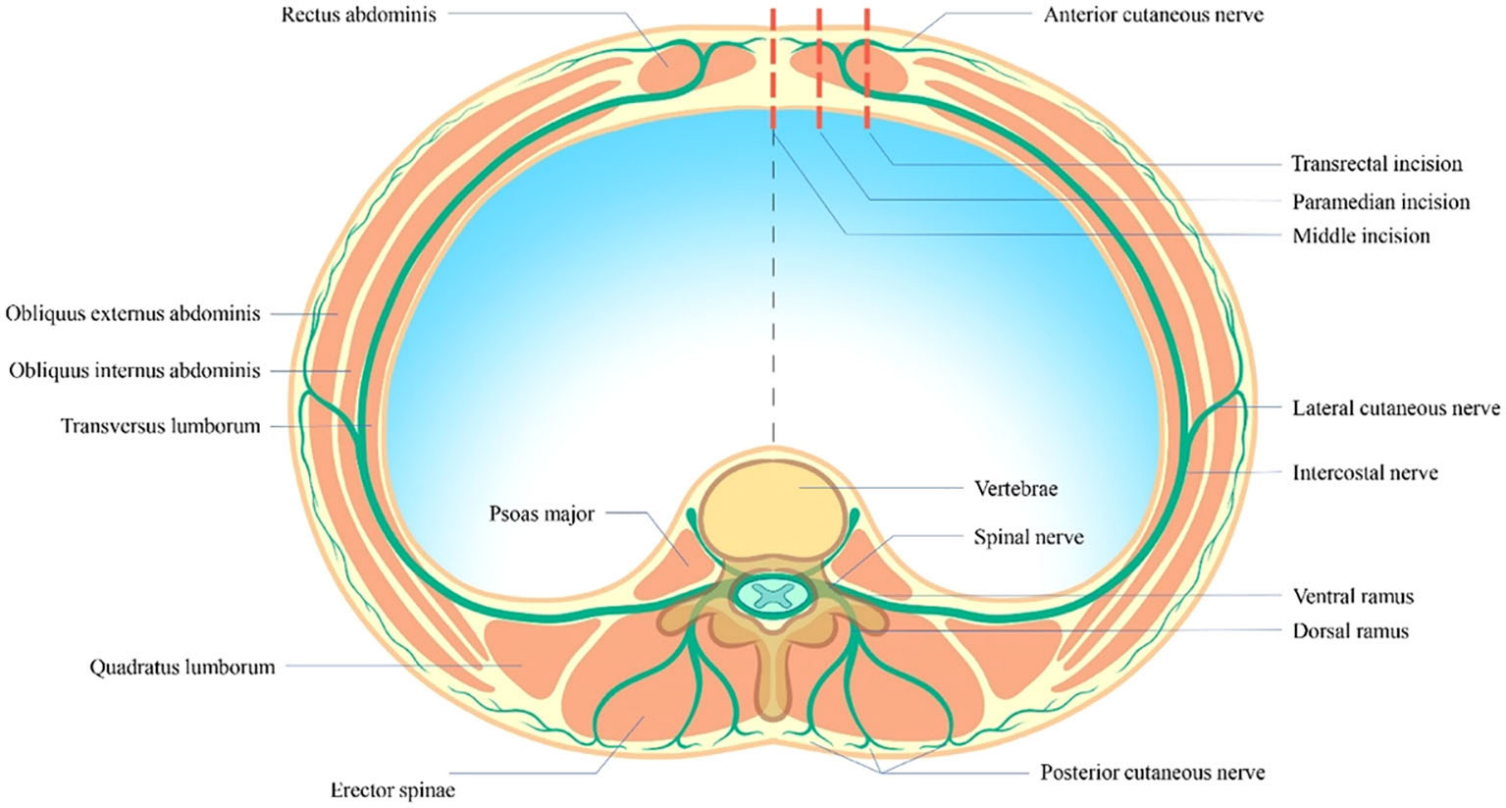
Various surgical incision paths cause distinct neuromuscular injuries.

In addition, three other potential explanations were also considered for the high prevalence of PPCs in the DAC group. First, complex surgical interventions inherently involve a greater degree of trauma and an extended period of anaesthesia and surgery, which consequently fosters an environment conducive to the development of PPCs. Compared with sigmoidectomy, ascending and descending colectomies entail intricate vascular trajectories, neighbouring organ interactions, and complex intestinal anastomosis techniques. Moreover, in comparison with the pelvic support surrounding the rectosigmoid colon, the absence of bony support in the DAC group further constrained the surgical field, thereby increasing procedural difficulty and correlating with a lower SRS score for evaluating surgical conditions in laparoscopic procedures in the presence of identical degrees of neuromuscular blockade (**Table 3**). Consequently, our study revealed that the surgical and anaesthetic times required for ascending and descending colectomy procedures are longer than those for rectosigmoidectomy. As reported in the literature, the incidence of PPCs is notably elevated in patients receiving extended anaesthesia and surgical procedures (8, 27, 78). Second, the surgical incision pathways for different CRC segments notably influence the development of PPCs (28). The ascending and descending colectomies typically necessitate paramedian or transrectal incisions (**Figure 3**); as a consequence, structures such as the anterior branches of the intercostal nerve, the rectus sheath (both anterior and posterior), and the rectus abdominis muscle are affected. In conjunction with compromised abdominal respiration, this impairment of rectus abdominis muscle function may accelerate the development of concomitant atelectasis and pneumonia. In contrast, procedures within the RSC group generally require a midline incision of the linea alba, exerting less impact on abdominal autonomy and breathing. Finally, as previously indicated, preoperative anaemia is an independent risk factor for PPCs (25, 27, 80). Likewise, relevant transfusions also promote the onset of this complication (81–83). Owing to the clinical characteristics of patients with DAC symptoms appearing later than those with RSC symptoms, anaemia (ASD=0.443) and transfusion (P=0.003) constitute a substantial proportion of patients with DAC symptoms (**Tables 2**, **3**); hence, anaemia is also a major cause of this postoperative complication.

Notably, cancer and inflammation are mutually reinforcing factors. CRC has been reported to induce inflammation through several mechanisms, with the expression of IL-1 being a mediator. Moreover, once the growth of a tumour exceeds its blood and nutrient supply, necrotic cell death in the tumour results in the release of various DAMPs, including IL-1 and HMGB1, both of which are related to lung inflammation (84). On the other hand, the immune microenvironment in the lung parenchyma can be altered by these DAMPs and establish a premetastatic niche for CRC (85). In summary, CRC patients may be at increased risk of developing lung injuries and PPCs because tumours elicit inflammation, and inflammation in the lungs promotes the metastasis of CRC.

Our investigation is subject to certain limitations. We endeavoured to curtail potential imbalances and limit the influence of clinical characteristics on PPCs by performing PSM for factors such as respiratory comorbidities, smoking status, and age. However, given the inherent peculiarities of these clinical variables in demographic and baseline data, achieving absolute balance remains an unrealistic expectation. Furthermore, although this study is based on the hypothesis that the steep Trendelenburg position induces more lung stress in the pathogenesis of pulmonary injury and PPCs, maintaining a sustained surgical position at one angle is impractical clinically because of the variety of surgical procedures performed, not even with prospective randomised controlled trials. Additionally, as a single-centre study with a population limited to a high-resource cancer centre, the generalisability of the results to other settings remains limited; hence, a larger sample size is needed. Despite these limitations, this study signifies progress in assessing the correlation between primary CRC sites, lung injury and the incidence of PPCs in patients who have received colorectal laparoscopy. Subsequent research could explore the impact of disparate surgical positions on the dynamic distribution of pulmonary ventilation, such as leveraging electrical impedance tomography (EIT) imaging. This approach could further elucidate the correlation between inclined surgical position, the distinct ventral and dorsal redistribution of tidal ventilation, and PPCs.

In summary, CRC patients display diverse surgical positions, procedures, and postoperative recovery patterns (75–77). Although the rectosigmoid colon with the Trendelenburg position in laparoscopy triggers epithelial lung injury, as indicated by elevated postoperative sRAGE levels, a lower PPCs incidence was unexpectedly observed than in those with tumours in the descending/ascending colon. Subsequent analyses confirmed that the primary CRC site was an independent risk factor influencing the onset of PPCs. The other baseline, clinical, and postoperative recovery characteristics cannot explain this effect. Therefore, not the Trendelenburg position and the resulting lung injury but rather the primary tumour site occurring in the descending/ascending colon directly affect the incidence of PPCs.

## Data Availability

The datasets contain identifiable contents of patients. The datasets studied in this research can be obtained from the corresponding authors upon reasonable request. Requests to access these datasets should be directed to TY, blizzardyt@163.com.
